# Mechanistic Pathogenesis of Endothelial Dysfunction in Diabetic Nephropathy and Retinopathy

**DOI:** 10.3389/fendo.2022.816400

**Published:** 2022-05-25

**Authors:** Jing Yang, Zhangsuo Liu

**Affiliations:** ^1^ Department of Ophthalmology, The First Affiliated Hospital of Zhengzhou University, Zhengzhou, China; ^2^ Research Institute of Nephrology, Zhengzhou University, Zhengzhou, China; ^3^ Henan Province Research Center For Kidney Disease, Zhengzhou, China; ^4^ Key Laboratory of Precision Diagnosis and Treatment for Chronic Kidney Disease in Henan Province, Zhengzhou, China; ^5^ Department of Integrated Traditional and Western Nephrology, The First Affiliated Hospital of Zhengzhou University, Zhengzhou, China

**Keywords:** diabetic retinopathy (DR), diabetic nephropathy (DN), diabetic endothelial dysfunction, CircRNAs, exosomes, cellular crosstalk

## Abstract

Diabetic nephropathy (DN) and diabetic retinopathy (DR) are microvascular complications of diabetes. Microvascular endothelial cells are thought to be the major targets of hyperglycemic injury. In diabetic microvasculature, the intracellular hyperglycemia causes damages to the vascular endothelium, via multiple pathophysiological process consist of inflammation, endothelial cell crosstalk with podocytes/pericytes and exosomes. In addition, DN and DR diseases development are involved in several critical regulators including the cell adhesion molecules (CAMs), the vascular endothelial growth factor (VEGF) family and the Notch signal. The present review attempts to gain a deeper understanding of the pathogenesis complexities underlying the endothelial dysfunction in diabetes diabetic and retinopathy, contributing to the development of new mechanistic therapeutic strategies against diabetes-induced microvascular endothelial dysfunction.

## 1 Introduction

The incidence of diabetes Mellitus is increasing dramatically worldwide. Epidemiological studies have shown that the incidence of diabetes will be expected to increase by over 50% by 2045 compared with 2017 (1). Diabetic diseases will be the 7th cause of mortality by 2030 in the world (1–4). Diabetic vascular complicating diseases are the primary cause of mortality in diabetes sufferers, the most common of which are diabetic nephropathy and diabetic retinopathy. As per the epidemiology investigation of T2D in PRC’s large cities, diabetic nephropathy and diabetic retinopathy account for 39.7% and 31.5% of diabetic microangiopathy, respectively (5). Current research reveals that diabetic microangiopathy (mainly refers to diabetic nephropathy and diabetic retinopathy) cannot be fully explained by the interaction between hemodynamics and metabolic factors or molecular modification in the state of high blood glucose. Clear evidence shows that its pathogenesis is multifactorial.

The coincidence of retina and kidney pathology in diabetic patients is well recognized, and certain literatures have proposed the definition of “renal-retinal syndrome” (6), in which microvascular endothelial dysfunction is an essential common pathological mechanism in the early development phase of the two diseases: microvascular endothelial cells are the main target of hyperglycemia damage. When the blood glucose concentration increases, the microvascular endothelial cells down-regulate the glucose transport rate, leading to intracellular hyperglycemia (7, 8) and a series of critical downstream pathways such as polyol pathway flux, elevated AGEs, elevated expressing of AGEs receptors and their activation ligands, stimulation of PKC subtypes and aminohexose signal path (9–11).

## 2 The Critical Role of the Endothelial Barrier in the Kidney and Retina

### 2.1 Glomerular Endothelial Barrier

The glomerulus barrier is the most intricate biomembrane, characterized by the permission of great water filtration rate, the unrestricted passing of small and medium molecules, and complete constraint of serum ALB and large protein (12–15). The glomerular basement membrane (GBM) and glomerular capillary endothelial cells, endothelial cell surface membrane, and podocytes constitute a glomerular filtration barrier (13, 14). GBM is a special basement membrane, mainly composed of a substrate and negatively charged proteoglycans. GBM comprises a fiber net involving IV-C, laminin, nidogen/entactin and heparin sulfate protein polysaccharides (15–18).

The GBM of a glomerulus capillary is about 240-370 nm thicker than that (40-80 nm) of other vascular beds (13–15). The glomerular endothelial cells are abnormally flat, and the circum-capillary ring height is 50-150 nm (14, 15). The fenestration area of glomerular capillary endothelial cells is enormous, accounting for 20-50% of the whole endothelium surface (16–20). The type IV collagen network is the framework of GBM (19–21). The basement membrane contains many proteoglycans, mainly attached to heparan sulfate chains, which are pivotal for the selective penetration of the barrier (22–25). Recent studies have confirmed that most of the valid charge density in glomerulus barriers is situated at the endothelium or epithelium layer. The occurrence of proteinuria precedes the morphological changes of podocytes in mice, indicating that GBM is a vital component of the glomerulus barrier (26, 27). On the lumen side, the vascular wall is covered with the ESL, comprising negatively charged glycosidoproteins, GAG, and membrane-related and excreted protein polysaccharides. This layer is involved in blood coagulation, angiogenesis regulation, rheology and capillary barrier (28).

### 2.2 Blood-Retinal Endothelial Barrier

The blood-eye barrier plays an essential role in protecting and maintaining the best visual cell function. It provides a greatly modulated chemical milieu for the intraocular non-vascular transparent tissue and serves as a drain to discharge waste from the metabolic activities of ocular tissue (29, 30). The blood-eye barrier includes 2 primary barrier systems: the BAB and the BRB (29, 30). Resembling the BBB, BRB regulates the transport of substances in the retinal capillaries, maintaining neural homeostasis, and prevents the leakage of macromolecules and other potentially harmful substances into the retina. The integrity of BRB is closely related to the structure and function of the retina (29, 30). Evidence indicates that BRB injury causes vascular permeability transition and is highly correlated to the pathophysiological process of many blinding kidney illnesses, like DR (29–31) and retina macula degeneration (30), etc.

BRB can be classified into inner BRB (iBRB) and outer BRB (oBRB). The internal barrier mainly comprises continuous RMECs and tight junctions (TJs) (32). The outer barrier comprises adjacent retinal pigment epithelium (RPE) cells and tight junctions (30, 31, 33). In the inner and outer barriers, cells are tightly connected to restrict fluids and molecules outside the barrier, and endothelial cells along with RPE cells actively regulate the discharge of fluids and molecules. Thus, although the amino acid or aliphatic acid in plasma fluctuates significantly, their concentration in the retina remains relatively stable.

Retinal vascular endothelial cells are single-layer cells covering the vascular cavity, acting as a selective barrier between the neural retina and blood circulation and providing nutrition and oxygen to the neural retina (29–31). Retinal vascular endothelial cell connections include tight junctions, adherence junctions (AJs) and gap junctions (GJs), which together form a complex to regulate cell permeability and maintain cell polarity to mediate cell adhesion and mutual communication and maintain the local microenvironment stability of the retina (32, 33). Among them, TJs is the most critical connection structure of BRB. The sound play of BRB’s normal function mainly depends on the TJs protein between vascular endothelial cells, including a variety of proteins such as transmembrane proteins Claudins, Occludins, membrane-related proteins (such as ZO-1, ZO- 2 and ZO-3) and TJs adhesion factor, etc. (32, 33). Moreover, pericytes, astrocytes, Muller cells and microglia in the retina exert a significant impact on the normal function of BRB (29–31). Muller cells extend from the subretinal space to the vitreous and act as anatomical and functional connection scaffolds between neurons and blood vessels, which can regulate the tension of blood vessels and maintain the integrity of BRB (34). Pericytes can regulate the local bloodstream and vasopermeability of retinal capillaries and support vascular endothelial cells. The loss of Pericytes will accelerate the destruction of BRB, increase vascular permeability, and lead to angiogenic macular edema (35).

## 3 The Effects of Endothelium Function Disorder on the Etiopathogenesis of DN and DR

### 3.1 Endothelial Dysfunction in DN

DN is the most commonly seen micro vascular complicating disease of diabetic sufferers. Its incidence is about 30% in T1D patients and 40% in T2D patients. In 1983, for the first time, Mogensen reported that changes in renal microvascular in diabetic patients could cause diabetic nephropathy, characterized by glomerular endothelial cell (GEC) damage, GBM thickening, and glomerular mesangial matrix proliferation and nodular glomerulosclerosis (36–41). In recent years, many reports have revealed that approximately 12-55% of ESRDs are due to diabetes (42–44). The opening of GECs and endothelial cells around the renal tubules is fairly significant for the selective penetration of the glomerular filtration barrier and the effective passage of large amounts of urine (45–47). GECs are lined with thick and negatively-charged filar glycocalyx, forming a network with glycosaminoglycans. The glycocalyx regulates vascular permeability and fluid balance and repels blood cells from the blood vessel wall. As DN progresses, a gradual decrease of the fenestrated endothelial surface can be observed in diabetic patients. In T2D sufferers, the decrease of glomerular endothelial pores is closely related to the loss of proteinuria and GFR (45–48). In terms of diabetes, hyperglycemia and its by-products can promote the growth of pro-oxidants and induce a pro-inflammatory environment featuring GEC dysfunction, which leads to proteinuria and renal fibrosis (41–44). GEC dysfunction is a multifactorial progression, which includes increased permeability of GEC, induction of endothelial cell apoptosis, glycocalyx decomposition, and impaired crosstalk between endothelial cells and other kidney cells (podocyte for instance) (40, 43, 44). GEC damage is attributed to the incidence of microalbuminuria, and this is an early event of DN (40). Microalbuminuria is also a sign of renal and systemic endothelial dysfunction (49, 50).

### 3.2 Endothelial Dysfunction in Diabetic Retinopathy

Since diabetes is becoming more common worldwide, DR is the most commonly seen and most serious ocular complication in many developed countries (9, 51–57). As one of the most active tissues in terms of metabolism, the retina is extremely sensitive to fluctuations in blood glucose levels. The pathological process of diabetic retinopathy consists of 2 stages: NPDR and PDR. As an early phase, NPDR is featured by PC loss in the retina capillary, which helps generate decellularized capillaries and improves microvascular penetrability as well as exacerbates endothelial BRB damage (9, 52–54, 57). Fibrovascular preretinal membranes, intravitreous hemorrhages, and amotio retinae may result from PDR, which is an advanced stage of retinal degeneration where fragile and tortuous new blood vessels form in the retina (9, 53–56). Hyperglycemia causes REC damage and the destruction of the blood-retinal barrier, resulting in the cumulation of exocellular fluids in maculas, as well as the capillary basement membrane thickening and a resulting ECM deposition (42–44). Due to the upregulation of angiogenesis factors (like VEGF), continual damage to the retinal microvasculature leads to retinal ischemia and capillary non-perfusion (45–50). Diabetic retinopathy not only impairs vision, but also elevates the risk of other life-threatening systemic vascular complications (40, 41).

DR pathogenesis involves many factors and approaches that lead to vascular dysfunction, especially endothelial dysfunction. As a result of hyperglycemia, retinal microangiopathy, inflammation, and neurodegeneration can occur, all of which can damage the blood-retinal barrier (BRB) and decrease blood flow, leukocyte stasis, and cause endothelial damage, increased vascular permeability, and pathological angiogenesis. It manifests clinically as decellularized capillaries and retinal angioedema (9, 51). As a result of hyperglycemia, retinal endothelial cells are damaged by ischemia, oxidative stress, and the release of pro-inflammatory factors. Damage to the blood-retinal barrier is now recognized as the mechanism.

BRB injury causes solute leakage and a rise in capillary pressure, which increase the osmotic pressure of the intercellular substance and lead to macular edema. DME is the primary cause of visual loss in diabetes sufferers, especially those with NPDR of T2D. In DME, there is vessel leakage, tissue edema, and complex exudate deposition in retinas. DR’s clinical symptoms appearing before persistent high blood glucose induces the loss of autoregulation in retinal cells and the tortuous expansion of arteries and veins (58–60). In diabetic retinopathy, retinal arteriovenous dilation raises hydrostatic pressure of capillaries, causing tissue edema *via* the Starlin rule, resulting in capillary wall dilation (microaneurysms) and rupture (hematomas), which are all typical symptoms of the condition (60). A reduction in capillaries may increase the production of VEGF and the rest of permeability factors, causing the rupture of BRBs and the extravasation of plasmatic protein into the interstitium. In addition to the severe leakage of DME, there are also slight variations in BRB in the early stages of PCDR (Pre-clinical diabetic retinopathy) (61). PCDR occurs earlier than clinical DR that threatens vision, which takes 8-15 years to show visible symptoms. PCDR is featured by PC loss, the basal layer thickening, and vascular retraction (62, 63). The increase in retinal vasopermeability in PCDR is featured by the existence of extravascular ALB, a decrease in the intercellular junctional complex, and an increase in the number of endothelial intracellular vesicles. In addition, PCDR is featured by diffuse loss of BRB without any formation of edema, as opposed to the large leakage of DME in the late stages of NPDR, and the diffuse increase in retinal vascular permeability is considered a key process that can further lead to NPDR (64, 65). In conclusion, a better understanding of the pathogenesis of BRB loss in the early and late phases of DR is helpful in the design of more valid DME therapies and in providing potential solutions to prevent DR from developing into NPDR.

## 4 Classic Regulators of the Endothelial Dysfunction of Diabetic Nephropathy and Diabetic Retinopathy

### 4.1 Cell Adhesion Molecule (CAM)

Studies have shown that CAMs are pivotal for the pathogenesis of vascular complications. Cellular proliferation, differentiation, the forming of cellular connections and the stimulation of white blood cells in inflammation sites, are some of the functions performed by these molecules (66–69). A variety of CAMs, such as ICAM-1, VCAM-1, and PECAM-1, can be found in the cell junction and are vital for the adhesion and migration of monocytes and lymphocytes to endothelial cells (66, 67). The soluble form of ICAM-1 is a marker for EC inflammatory events and damages. PECAM-1 is pivotal for maintaining the integrity of vessels and the barrier function of endothelial cells (68). It has been demonstrated that IL-1, TNF-β, VEGF, and NF-κB can stimulate the expressing of ICAM-1 and VCAM-1 in endothelia (68, 69).

In diabetic patients, the elevated level of AGEs and ROS can activate leukocytes and increase NF-κB transcription in endothelial cells (70–73). NF-κβ also increases the expressing of ICAM-1 in high glucose (74). The increased VCAM-1 expression and ICAM-1 expression in high glucose has proven to be directly associated with the occurrence and development of DN. An increase in VCAM-1 expression has been observed in diabetic mouse models. According to cross-sectional studies, VCAM-1 circulating level and ICAM-1 circulating level in diabetes sufferers with nephropathy are higher than those in patients without renal injury (75, 76). VCAM-1 circulating levels are associated with proteinuria (76). Elevated plasma levels of ICAM-1 in T1DM patients are independently correlated with the occurrence of continuous microalbuminuria (77–84). The interaction between ICAM-1 expressed by GECs and LFA-1 is thought to be essential for migrating T cells to the kidney (78). ICAM-1 deficient db/db diabetic mice displayed a diminished ability to homing to the glomerulus as compared with ICAM-1 sufficient db/db diabetic mice (85).

Numerous evidences suggest that inflammatory events are vital for the etiopathogenesis of DR (71, 86). Furthermore, NF-κB activation promotes the inflammatory cascade in the vitreous humor and serum, thereby upregulating ET-1 and downregulating eNOS, causing REC damage, capillary stenosis, retinal ischemia, and abnormal blood flow. An activated form of NF-κB also overexpresses ICAM-1, leading to REC damage and the destruction of the blood-retinal barrier (87, 88). ICAM-1 and VCAM-1 are regulated upward in the conjunctival tissues of diabetic sufferers (71, 86, 89), and ICAM-1 is capable of acting synergistically with RAGE to realize the mediation of white blood cell recruiting in the event of acute inflammation (90). In addition to VEGF, studies have shown that there is a positive association between the sera ICAM-1 level and the degree of damage in the external limiting membrane (ELM) (91). Hence, supervising the level of serum dissolvable VCAM-1 in diabetic sufferers might contribute to evaluating the seriousness and activities of DR (92, 93).

### 4.2 VEGF Family

VEGF belongs to the super gene family of VEGF/PDGF, and the most widely studied member is VEGF-A (94–99) that comprises 8 exons. Utilizing variable splicing, at least 6 diverse isoforms are produced, which can be differentiated by AA lengths, including VEGF_121_, VEGF_145_, VEGF_165_, VEGF_183_, VEGF_189_ and VEG_F206_, among which the most affluent VEGF-A isoform in humans is VEGF_165_ (94, 99). VEGF-A is especially vital for the differentiation, proliferation and survival of endothelial cells of the vasculature, promoting endothelium-dependent vasodilation and raising vascular permeability (94, 99). VEGF-A triggers endocellular signal transduction by connecting to either VEGFR-1 or VEGFR-2, and the coreceptors NP1 and NP2. VEGFR-2 is accountable for the mediation of the majority of VEGF-A biology roles at normal status (96–99).

In kidneys, VEGF-A is expressed mainly by glomerulus foot cells, and VEGF-A mRNAs can be detected as well in the distal tubule and collecting duct (100–103), while VEGFR-2 is expressed by GECs and pericapillary ECs, as well as cortex interstitial fibroblasts and renal medulla interstitial fibroblasts (104–107). Therefore, the typical VEGF signal transduction in the glomerulus can be considered as the process where podocytes secrete VEGF-A and then pass through the filter barrier contrary to urine flow and bind to VEGFR-2 expressed by glomerular endothelial cells surface (104–107). The expressing of VEGF-A by foot cells is imperative for healthy renal development. Studies have revealed that there is a paracrine VEGF/VEGFR2 regulatory loop between GEC and podocytes (105). The overall loss of VEGFR2 leads to significant kidney abnormalities and glomerular microvascular system deficiency. VEGF generated by podocytes modulates the structure and functions of adjacent endothelial cells. Experiments have shown that both the expression of VEGF and VEGFR2 in kidneys increase in the early phase of DKD (106, 107), and the inhibition of VEGF-A or VEGF receptor in diabetic animals can prevent proteinuria and alleviate glomerular damage (106, 107). Although the activation of VEGF/VEGFR in the early phase of DKD can lead to novel blood vessel formation and other glomerular damage, the loss of podocytes in the later stage induces a decrease in VEGF signal, which triggers sparse blood vessels and renal fibrosis. Recent studies have indicated that the usage VEGF antagonists can protect kidneys of most diabetic rodents (104, 107), while for cancer patients and some non-diabetic nephropathy rodents, if they receive anti-VEGF treatment, their kidneys will potentially be damaged (108).

In the etiopathogenesis of DR, VEGF is considered a vital vascular growth factor that is implicated in pathological retinal neovascularization and increases vascular permeability (61, 109–119). The majority of literature reports and clinical data demonstrate that VEGF is increased in the vitreous of PDR patients (61, 113–119). In addition to endothelial cells, other retinal cells can also produce VEGF when activated or stimulated continuously by high glucose levels, like RPEs, PCs, Müller cells, stellate cells, and glial cells (117, 118). VEGF is vital for the modulation of ocular angiogenesis and vascular permeability. In the vitreous humor and fibrovascular tissue of PDR eyes, elevated levels of VEGF can be observed (61, 119). VEGF levels in the serum and vitreous are related to blood glucose control in diabetic patients (119). There is a significant association between the increase in VEGF contents in the vitreous body and the severity of DR (61, 119). Diabetes-induced increases in the level of VEGF are considered a biomarker of DR severity. Experiments have revealed that intravitreal injection of VEGF can produce various symptoms of NPDR and PDR: nonperfused capillaries, vasodilatation, and tortuous arterioles characterized by endothelial hyperplasia and microaneurysms (61, 118, 119). The degree of damage to the external limiting membrane (ELM) is correlated positively with the level of serum VEGF, which suggests that the level of VEGF is related to the severity of DR and the degree of damage to the external limiting membrane. VEGF regulates DR-related inflammation in the early phase (118). There might be a correlation between the elevated stimulation of NF-*B in NPDR as well as PDR sufferers and the increased expression of VEGF (119). VEGF induces the expression of MCP-1, IL-8, TNF-α and ICAM-1 in retinal endothelial cells *via* stimulating the NF-kB pathway (61). VEGF boosts the adhesion of leukocytes to the blood vessel wall by enhancing the expressing of ICAM-1 and VCAM-1 of ECs (61, 117). Furthermore, VEGF might promote early diabetes retina white blood cell adhesion in retina arterioles by upregulating the expressing of ICAM-1 (118). It has been reported that an increase in serum VEGF levels can stimulate the formation of ROS, resulting in endothelial activation (119).

The PDGF family includes PDGF-A, PDGF-B, PDGF-C, and PDGF-D. PDGFs are secreted by endothelial cells, macrophages and epithelial cells (120–122). PDGF-C is more structurally similar to VEGF than PDGF-B, while VEGF shares approximately 25% sequence similarity to all PDGFs (123). PDGFs exert their diverse functions under physiological or pathological conditions by binding to platelet-derived growth factor receptors (PDGFRs) (124, 125). PDGF is involved in the embryonic development of many organs, including the brain, lung, retina, vasculature, and kidney, of which the most interesting is the role of PDGF in the vasculature (124–128). PDGFs play an important role in physiological angiogenesis by recruiting vascular endothelial cells, a large number of studies have shown that PDGFs and their receptors play a key role in many ocular neovascular diseases, such as proliferative diabetic retinopathy (PDR), retinopathy of prematurity (ROP), and neovascular age-related macular degeneration (NVAMD) (122, 128). Three isoforms of PDGF (AA, AB, and BB) have been shown not only to be involved in the process of neovascularization in PDR, but also to play an important role in the formation of fibrotic tissue in the retina of PDR patients (128). The concentration of PDGF-AB in the vitreous humor of patients with diabetic retinopathy is increased, and PDGF-C and PDGF-D are also abundantly expressed in the retina (128). Recent studies in ocular neovascularization models have shown that simultaneous inhibition of VEGF and PDGF, especially PDGF-B, enhances antiangiogenic effects (122–125). PDGF is also upregulated in diabetic nephropathy, and both imatinib and PDGFR-β gene knockout attenuated renal injury, especially mesangial dilation, in different diabetic nephropathy mouse models (129–132). Notably, PDGF has been extensively reported to play a beneficial repair role in diabetic foot and high glucose-induced endothelial progenitor cell injury, rather than the negative role of PDGF currently reported in DR or DN (126, 127).

### 4.3 Notch Signaling

The Notch signaling pathway is a highly conserved cellular signal transduction system present in most multicellular organisms. Signals are transmitted between adjacent cells through Notch receptors, which can regulate cell differentiation, proliferation and apoptosis (133–135). The Notch receptor is a single transmembrane protein encoded by the Notch gene, which is a hetero-oligomer composed of a large extracellular part, which contains a short extracellular domain through calcium-dependent non-covalent interaction with a short extracellular domain (136). Notch protein interaction between the transmembrane domain and a short intramembrane domain. Four Notch receptors (Notch 1, 2, 3, 4) have been found in mammals including humans (137). Notch ligands are also single-transmembrane proteins expressed on the cell surface, and adjacent cells transmit Notch signals through the binding of Notch receptors and ligands. There are five Notch ligands in the human body, namely Jagged 1, Jagged 2, Delta 1, Delta 3 and Delta 4 (137). After Notch ligand binds to the receptor, the activation of Notch signal is triggered, and the Notch receptor undergoes two proteolysis successively and is further transferred to the nucleus, thereby activating the transcription of target genes and exerting biological functions (138). The role of the Notch pathway as a master regulator of angiogenesis is well established, and Active Notch provides a pro-survival, anti-inflammatory and anti-atherosclerotic environment that reduces adhesion molecules such as ICAM-1 and VCAM-1 (139). Expressed to maintain endothelial integrity by participating in the formation of endothelial junction complexes (139).

In the mouse retinal vasculature, active Notch1, Jag1, Dll1 and Dll4 have been described with distinct distribution patterns (140). Cellular analysis and gene inactivation or mutation of mouse or zebrafish retinas, combined with ocular disease models, have demonstrated that Notch signaling plays an important role in embryonic and postnatal ocular angiogenesis and is involved in angiogenic ocular diseases (141). Recent findings show that NOTCH1 signaling in retinal microvascular endothelial cells induces vascular permeability in diabetes: NOTCH1 ligands JAGGED1 and DELTA LIKE-4 are secondary to hyperglycemia and activate the canonical and rapid atypical NOTCH1 pathways, ultimately disrupts adherent junctions between endothelial cells by causing diabetic endothelial dissociation (142). Additionally, modulation of Notch signaling enhanced neovascularization and reperfusion in diabetic mice by modulating EC responsiveness to VEGF. In PDR, aberrant Notch signaling may interact with VEGF signaling at multiple levels to mediate retinal microvascular endothelial cell dysfunction (143).

The vast majority of research on Notch signaling in diabetic nephropathy has focused on the effects of Notch signaling on podocytes and tubular epithelial cells (144–149). Although no studies have directly assessed the role of Notch signaling on renal arteriole function or neovascularization in diabetic nephropathy, Notch signaling does inhibit diabetic extrarenal angiogenesis by altering the sensitivity of hemangioblasts to VEGF (150, 151). Although its effect on glomerular endothelial cells in diabetic nephropathy remains to be further studied, the role of Notch signaling in microvascular endothelial cells in diabetic retinopathy and diabetic ulcers has been confirmed (152, 153).

### 4.4 ROS

ROS are oxygen-derived, highly reactive molecules, including free radicals such as superoxide (O 2-) or hydroxyl (•OH) and non-radical ROS such as hydrogen peroxide (H_2_O_2_) (154). ROS have physiological roles in cell signaling related to cell proliferation and survival, and are tightly regulated and balanced by cellular antioxidant responses (155). There is increasing evidence that ROS from ECs and other cell types such as vascular smooth muscle cells, myeloid cells such as neutrophils and macrophages can also stimulate angiogenesis (155). There are many sources of ROS, including NADPH oxidase (NOX), mitochondrial electron transport chain (ETC), xanthine oxidase, unconjugated endothelial nitric oxide synthase (eNOS), cytochrome P-450 oxygenase, and cyclic oxidase (156). Vascular NOX isoforms (Nox1, Nox2, Nox4, and Nox5) differ in activity and cell specificity in response to agonists, growth factors and hypoxia, and the types of ROS released upon activation (157). In ECs, ROS originate from NOX (especially Nox2 and Nox4) in the plasma membrane or the intracellular cytoplasmic compartment, as well as mitochondria, and play a key role in the angiogenic response induced by growth factors such as VEGF (158). Excessive ROS can lead to oxidative stress, which can lead to various cellular changes, which can lead to organ dysfunction and trigger diseases such as cancers, atherosclerosis and diabetic microvascular complications (159).

ROS play a direct role in diabetic nephropathy. In GECs, hyperglycemia saturates glucose metabolism and leads to activation of deleterious pathways, such as the polyol pathway, hexosamine pathway, AGE/RAGE axis, and PKC pathway, leading to overproduction of endogenous ROS (160). Oxidative stress and inflammation and their interactions are considered major pillars of CKD pathogenesis and progression. Oxidative stress promotes inflammation through the formation of pro-inflammatory oxidized lipids, AOPPs, and AGEs (advanced glycation end products), while activation of nuclear factor kappa B (NFκB) transcription factors in a pro-oxidative environment promotes the expression and recruitment of pro-inflammatory cytokines (161). Activation of leukocytes and other resident proinflammatory cells (161). Likewise, pro-inflammatory cytokines, such as tumor necrosis factor-α (TNFα), bind to their receptors on glomerular endothelial cells and trigger signaling pathways that activate the nuclear factor kappa B (NF-κB) transcription factor (162). In addition, in the context of chronic inflammation, activated leukocytes produce ROS, chlorine and nitrogen, thereby exacerbating and perpetuating oxidative stress (162). Indeed, initial ROS-mediated renal injury triggers subsequent renal and systemic inflammatory responses. Thus, in a cohort of 176 patients with CKD stages 1 to 5, serum levels of hs-CRP, interleukin-6, and malondialdehyde were significantly elevated and inversely correlated with GFR, while serum SOD and glutathione peroxidase levels were significantly reduced (161). Notably, IL-6 and hs-CRP were positively correlated with malondialdehyde, and with superoxide dismutase and glutathione peroxidase negative correlation, further supporting the relationship between inflammation and oxidative stress in CKD (161). One of the most important functions of the endothelium is the secretion of nitric oxide (NO), a relatively unstable diatomic free radical involved in a variety of biological processes, including in smooth muscle cells mediated by cyclic guanosine monophosphate (cGMP) vasodilation, inflammation and immune response (163). The relationship between NO and ROS is bidirectional, low levels of NO in the endothelium induce the expression of antioxidant genes and protect renal endothelial and mesangial cells from apoptosis and fibrosis, while on the other hand, increased ROS levels reduce Endothelial cells generate NO through inhibition and/or uncoupling of NOS enzymes (164).

Oxidative stress is the main pathogenic cause of DR, and oxidative stress can damage the integrity of cell membranes, induce apoptosis, microvascular damage and barrier damage, and ultimately lead to the development of DR (165). Polyol pathway activation represents one of the processes observed under conditions of hyperglycemia-induced oxidative stress during the pathogenesis of DR, and this pathway is also known as the sorbitol-aldose reductase pathway (165). Excessive activation of the polyol pathway leads to the accumulation of ROS, which induces oxidative stress in cells (165). Sorbitol and fructose accumulate intracellularly, leading to increased osmotic pressure, edema rupture, and impaired membrane permeability (166). The hexosamine pathway may also mediate the toxic effects of ROS in hyperglycemia. In the presence of elevated glucose levels, large amounts of ROS are produced, which may inhibit the activity of glyceraldehyde-3-phosphate dehydrogenase (GAPDH), resulting in the influx of glycolysis products into the hexosamine pathway (167). Glucosamine production from activated hexosamine increases H2O2 production, which further leads to increased oxidation, cellular endothelial changes, increased vascular permeability, and angiogenesis. Inhibition of GAPDH also induces the activity of the AGE pathway through interaction with intracellular methylglyoxal, leading to increased retinal oxidative stress (167). Activation of the PKC pathway can lead to endothelial cell damage by increasing endothelial permeability, altering NO bioavailability, reducing prostaglandin production, inducing VEGF expression, and inducing thromboxane and endothelin-1 (ET-1) production. The hyperglycemic state induces the accumulation of ROS and the synthesis of diacylglycerol (DAG), leading to activation of the PKC pathway (31). PKC pathway activation alters NO production through eNOS expression, directly affects vascular tone and permeability, and ultimately promotes endothelial dysfunction (31). The receptor for AGEs (RAGE) also plays an important role in DR pathogenesis, as its activation mediates a wide range of biological effects, including increased ROS levels, cytokine release, and altered cellular function and death (168). AGEs and RAGEs accumulate in retinal microvessels and interact directly with intracellular proteins, leading to endothelial dysfunction (168). Additionally, during the pathogenesis of DR, ANG-II induces vasoconstriction, inflammation, oxidative stress, cellular dysfunction, angiogenesis, and fibrosis, and can activate NADPH enzyme levels, thereby increasing ROS production and directly damaging endothelial cells (169).

## 5 Research Hotspots of the Endothelial Dysfunction of Diabetic Nephropathy and Diabetic Retinopathy

### 5.1 Exosomes

Exosomes were initially found in the supernate of RBCs of *in vitro* cultured sheep. Exosomes, 40-100 nm (diameter) and 1.10-1.18 g/ml (density), are vesicle-like bodies that are actively secreted by cells (170–175). In 1996, Raposo found that B cells can promote T cell proliferation and inhibit tumor growth by releasing exosomes capable of expressing major histocompatibility complex (MHC) molecules (170). Exosomes are capable of carrying various protein, mRNAs, miRNAs, circRNAs, and participate in cellular interaction, motility, angiogenesis, and oncocyte proliferation (170–175). The intracellular lysosomal particles invaginate and form multivesicular bodies. When stimulated, the multi-vesicular bodies fuse with the cellular membrane and secrete out of the cell vesicles with a uniformed size at 40-100 nm in diameter. These vesicles are exosomes (171, 173–175). The formation and release of exosomes involve endosome sorting transporters and some related proteins such as CD9 and CD63 (170–172). When circulating in the blood, exosomes can act as shuttle vectors or signal transducers at their primary site and at a certain distance from the primary site (170, 172, 173). Recent studies have discovered that the exosomes in the circulation and humor of T2D angiopathy sufferers exhibit a different amount of protein and RNA content compared with that of healthy subjects, which can be taken as potential diagnostic markers and ingredients to deliver reversed high glucose damage, such as the protective role that exosomes are releasing miR-216 plays in the process of atherosclerosis through cell-to-cell communication (174–176).

Normally, exosomes can be excreted by renal cells, like podocytes, renal tubular cells, GECs, and glomerular mesangial cells (177–184). Numerous studies have indicated that changes in culture conditions may influence the composition of exosomes. For instance, under the pressure conditions of hypoxia, acidic pH, uremic toxins, high glucose, and oxidative stress, the number of exosomes increases and their composition changes (177–182). Exosomes can induce the release of cytokines and promote the aggregation of inflammatory cells. Damaged renal cells could secrete exosomes that can be transferred to other normal renal cells, altering the phenotype of normal kidney cells, and promote cell-to-cell interaction. The urinary exosome is a new biomarker for diagnosing DN (177–181). Exosomes significantly improve renal function and significantly enhance LC3 and Beclin-1 (179). As compared to healthy controls, the urine exosomes of DN patients show a new array of protein changes, including MLL3, AMBP, and VDAC1 (181). There is evidence that serum exosomes from diabetic db/db mice severely impair the endothelial function of non-diabetic db/m + mice. Proteomic analyses have revealed that arginase 1 (Arg1) is increased significantly, and it has been proven that serum exosomes deliver Arg1 to endothelial cells (185). In STZ-induced diabetic nephropathy rats, platelets with elevated CXCL7 expression can activate the mTORC1 pathway and result in glomerular endothelial dysfunction (186).

Studies have confirmed that intravitreal injection of exosomes derived from mesenchymal stem cells can ease DR (187–189). PPARγ is an active component of exosomes. A study demonstrated that in patients with proliferative DR, PPARγ levels was significantly increased in both aqueous humor and vitreous humor, revealing a vital effect of exosomes on DR. Various researches have tested the effects of exosomes on diabetic microangiopathy. Researchers found that exosomes in the plasma of DR patients increased the expression of cytokines and angiogenic factors, indicating that circulating exosomes might play a crucial role in carrying inflammatory factors (190, 191). A recent study has demonstrated that high glucose levels affect the anti-angiogenic exosomal miRNA levels (192). In a study on diabetic microvascular complications, exosomes that carry circRNA- cPWWP2A could non-directly regulate microvascular EC function (193). Studies have demonstrated that plasma exosomes mediate hyperglycemia-induced retinal endothelial damage through up-regulation of TLR4 signaling pathway (194). In previous researches, investigators demonstrated that circulating miR-15a contributed to the pathogenesis of diabetes and induced oxidative stress and cell damage *via* exosomal transport (195).

### 5.2 Circular RNA (circRNA)

CircRNA is a class of non-coding RNA (196–199) without a 5’end cap and a 3’end poly(A) tail, presenting a covalent bonding round architecture. circRNA can be distinguished from traditional linear ribonucleic acid. It has an enclosed helical structure and exists widely in the eukaryotic transcriptome. Most circRNAs comprise exon sequences, conservative in diverse species, and display expression overlaps in tissues and different developmental phases (197). Because of its insensitivity to nucleases, circRNA is more stable than linear ribonucleic acid, which makes it stand out in the development and application as a new clinical diagnostic marker (198, 199). CircRNA has been found to be abnormally expressed in many illnesses line malignancies, vascular illnesses, inflammatory illnesses, and neurological illnesses. CircRNA displays a diverse expression in angiogenic extreme vascular diseases, and the abnormal expression of circRNAs is related to the incidence and development of diabetic vascular complications (200, 201).

Studies have demonstrated that in HUVEC induced by HG, the level of circRNA-001175 is significantly down-regulated, and the up-regulation of circular RNA-001175 can prevent cell apoptosis induced by high glucose in HUVEC (202). In the following research, HG increased the expressing of circRNA 0054633 in HUVEC, and its down-regulation further deteriorated the endothelial cell dysfunction caused by high glucose (203). Studies have revealed that circular RNA 0054633 can protect endothelial cells from damage stimulated by high glucose through targeting the miR-218/HO-1 axis (203). Moreover, the expressing of circRNA HIPK3 was down-regulated in HG cultured HUVEC, and the overexpression of lentivirus-mediated HIPK3 inhibited HG-triggered EC death and programmed cell death *via* targeting miR-124 (204). In high glucose treated HUVEC, the circRNA 0068087 level is regulated upward. The knockout of circRNA 0068087 inhibits HG-triggered EC inflammation/NF-κB/NLRP3 inflammatory body signal path *via* the inactivation of TLR4 (205). These findings have shown that there is great potential for circRNAs to be studied as a diagnostic basis for HG-associated EC function disorder or predictive biomarkers.

Researches have recently highlighted the role of circRNA in the DN, while current evidence suggests that circRNA as a miRNA sponge plays an important role in its pathophysiology. CircRNA-15698 could increase TGF-β1 protein in DN and stimulate the synthesis of ECM-related proteins. the knockdown of circRNA-15698 could decrease the fibrillar proteins (206). In addition, a recent study has demonstrated that circLRP6 sponges miR-205 and controls mesangial cell damage in high glucose conditions (207). CircRNA has been found to affect the regulation of inflammatory events in other organs’ endothelia *via* consistent targets in the kidney, such as TGF-β1, which indicates that circRNA may be the key regulator in DN pathogenesis (208).

Circ_0005015 have been found to be significantly increased in both vitreous humor and plasma, and can regulate HRMECs proliferation by acting as the sponge of miR-519d-3p (209). CircHIPK3 overexpression enhances microvascular leakage and the number of acellular capillaries in diabetic retina blood vessel function disorder by regulating HRMECs (210). In diabetic conditions, c-myb can activate the axis of circHIPK3-miR-30a-3p-VEGFC, which affects the retinal microvascular endothelial function (210). In the DR model, the deficiency of cZNF609 inhibits pathological angiogenesis (211). In contrast, the circDNMT3B concentrations in FVM and human retinal microvascular EC (HRMEC) are decreased by high glucose treatment. CircDNMT3B targets MiR-20b-5p which actively regulates endothelial angiogenesis by regulating the expression of BAMBI (212). The findings of these studies further demonstrate the importance of circRNAs in the pathogenesis of diabetic retinopathy.

### 5.3 Interaction With Neighboring Cells

#### 5.3.1 Crosstalk Between Renal TECs and GECs

Several studies have demonstrated that glomerulo-tubular balance and tubule-glomerular feed-back influence the development of DKD (213–218), on the foundation of which new hypoglycemic drugs could increase glucose in urine excretion, regulate BG, and thus decrease glomerulus damage in DKD patients (219). VEGF, Ang-1 and inflammation factors are abnormally secreted in DKD (220–224). Furthermore, damaged GECs produce cytokines, exosomes, and *via* autophagy, they induce TECs damage featuring structural changes and altered functional characteristics (225–231). Since TECs and GECs are located at a distance, their abnormal crosstalk is instrumental in the development of DKD. Microalbuminuria can be seen in early DKD because of the changes on GEC surface, including its component glycocalyx (232–234). Recent studies have shown that the paracrine communication between GECs and TECs can contribute to DKD. Recent proofs have revealed that glomerulus filter barriers and renal tubular interstitial compartments are in kinetic equilibrium, in which different types of cell damage can affect each other and result in tissue dysfunction (235). Genetic and protein expression profiles indicate that changes in diabetic glomeruli affect a number of metabolic and signaling pathways, either in individual glomerular cells or in the context of their mutual interaction (236). TECs under high glucose and proteinuria conditions induce cascade inflammation *via* autocrine or paracrine secretion of cytokines, miRNAs, and extracellular vesicles. Inflammatory factors cause apoptosis, necrosis, and trans-differentiation of the glomerular vascular network, causing damage to the architectures and functions of GECs. The impaired GECs induce low blood supply in the kidney tubules, thereby exacerbating the damage to TECs.

Ang-1 and Ang-2 play a vital role in mature blood vessel formation (216, 220). TECs generate Ang-1, which bind to the tyrosine kinase 2 (Tie-2) spot of GECs and reduce the endothelial function of GECs (216). VEGF is secreted by TECs and binds to VEGFR expressed by GECs, generating a VEGF/VEGFR axis, which controls the structure and function of GECs (223). The expressing of HIF-1α in TEC’s is increased, and HIF-1α enters nuclei and binds with the HIF-1β subunit to generate HIF-1, which stimulates down stream inflammation responses to induce GEC autophagy and programs cell death (236). As TECs accumulate in dense plaques, they regulate the contraction and relaxation of the glomerulus arteriole and affect GFR (237). SGLT2 expressed by proximal TECs decides the re-absorption of kidney BG (238). In DKD sufferers, SGLT2 is overexpressed due to the presence of more glucose molecules (238, 239). The SGLT-2 inhibitor can straightly decrease great GFR through the repairment of tubule-glomerular feed-back (240).

#### 5.3.2 Crosstalk Between Pericytes and Retinal Endothelial Cells

Two major cellular components of retinal microvessels are endothelial cells (EC) and pericytes (PC). Both types of cells share the same basement membrane on the blood vessel wall. ECs and PCs are closely connected by intercellular space and communicate with other signaling factors (such as cytokines and extracellular exosomes). Interactions between these two types of cells occur in areas rich in extracellular matrix (ECM) (241–245). There are several factors affecting the mutual effect between ECs and PCs in pathological state, including changes in advanced glycation end products, increased leukocyte adhesion and inflammatory cytokines (246, 247).

It has been demonstrated that the inhibition of VE-PTP helps stabilize the ocular vascular system and prevent retinal and choroidal NV (248, 249). However, Tie2 can also be expressed by pericytes, and its signal transduction in pericytes is critical for pericyte migration, endothelial sprouting and neovascularization (248, 249). Studies have discovered that the coverage of pericytes is reduced by producing transgenic mouse strains, while inadequate pericyte coverage can lead to BRB damage and increased leakage of the developing retinal vascular system, thus impairing visual performance (250).

## 6 Treatment of Diabetic Nephropathy and Diabetic Retinopathy

### 6.1 Treatment of DN

Diabetes complications, especially DKD, can be prevented if long-term and intensive blood glucose control can be monitored from its early stage. However, intensive glycemic control in diabetic patients with pre-existing diabetic complications fails to reduce the risk of DKD progression (251–254). The studies on T2D patients and early CKD patients revealed that compared with standard treatment, all-cause death and cardio-vascular death risks for patients undergoing intensive blood glucose control were 30% and 40% higher. Long-term intensive blood glucose control can lead to hypoglycemia and cast negative impacts on curbing cardiovascular illnesses or all-cause death (251, 252). The National Kidney Foundation’s KDOQI and the KDIGO guide lines suggested that the HbA1c target value should be controlled at approximately 7.0% to avoid or postpone the development of diabetic micro-vascular complicating diseases, except for individuals suffering the risk of low blood glucose, like diabetic diseases and CKD (252).

For the treatment of high blood pressure, the JNC-8 suggests that drug intervention should be started when SBP exceeds 140 mmHg or DBP exceeds 90 mmHg with the aim to lowering these levels (255). In common patients with hypertension, including diabetes patients, initial antihypertension therapy might involve thiazide diuretics, calcium channel blockers, ACE inhibitors, or ARB. The KDIGO guidelines recommend that ACE or ARB should be used in all patients with CKD and proteinuria with a target of blood pressure lower than 130/80 mmHg. Evidence has unraveled that using ACEI or ARB to block the renin-angiotensin system can curb the development of DKD in sufferers with large albuminuria, whereas it may have the side effects of hyperkalemia and AKI (252).

Although at present there are methods for managing diabetes and hypertension using ACEI and ARB, DKD still has a large residual risk. New drugs with targeted causal links, like glomerulus hyper-filtration, inflammatory events, and fibrotic activities, have always been the main hot spot in terms of the design of novel therapies. Promising drugs include Rubista (PKC-β suppressor), Baritinib (selective JAK-1 and JAK-2 inhibitors), Pentoxifylline (antiinflammation and antifibrosis), Atrasentan (selective ETAR antagonist), and fennel one (remarkably selective non-steroidal MRA) (251). However, up till now, there is no phase 3 clinic information of those drugs yet, and no drugs have been approved for DKD. The SGLT2 suppressor, empagliflozin, can remarkably reduce mortality as well as hospitalizations for heart failure. The analyses of prespecified secondary results revealed that empagliflozin curbed the development of DKD and reduced the incidence of clinical correlation renal results in CKD phases 2-4 patients (256). In recent years, a number of randomized controlled experiments have confirmed the great role of SGLT−2 inhibitors in renal protection, suggesting that SGLT−2 inhibitors can become a new hope for improving the prognosis of DKD patients, but the mechanism of renal protection warrants further studies (256, 257). The therapeutic effect of non-steroidal mineralocorticoid receptor antagonists on DN patients is also one of the currently popular study fields (258, 259).

### 6.2 Treatment of Diabetic Retinopathy

Laser photocoagulation is still the primary treatment for DR that threatens vision (165, 260–266). Nevertheless, although timely and appropriate laser treatment can significantly prevent vision loss, the destruction nature of lasers is related to remarkable adverse effects. In addition, sufficient laser treatment cannot guarantee the reversal of vision loss. For that reason, investigators persistently seek novel and more valid treatments to improve vision without damaging tissues. There are two laser treatments for DR: the PRP for PDR and the macula laser photocoagulation for DME. The main function of PRP is to perform laser burning on the whole retina, preserving the central macula to facilitate the regression of retinal neovascularization and prevent its development. Although it has an undisputed effect in preventing severe vision loss, PRP is usually related to many adverse effects, like difficulty in adapting to light darkness (25%), tiny loss of vision (10%), and loss of peripheral vision (5%), color vision alteration and macular edema worsening (260, 263, 265). Besides, the results of DRS and ETDRS indicate that DR sufferers in milder phases may not benefit from laser treatment (267).

Vitreous body resection has always been the main surgical therapy for advanced retinopathy, which includes two blinding complications: chronic vitreum hemorrhage and TRD (165, 260–263). It is a double-edged sword for diabetic eyes. Although it decreases the risk of retinal blood vessel neogenesis and macula edema, it also elevates the risk of iris blood vessel neogenesis and cataract forming (260). In clinical studies of DR vitrectomy, for T1D sufferers with severe vitreous hemorrhage, those underwent vitrectomy (occurred within 1-6 months) were more likely to achieve better eyesight (261, 262) than those received vitrectomy (occurred for 1 year). However, the benefits of this operation have not been reported in T2D sufferers, which might be related to more severe macular ischemia in these patients. Vitreous body resection is also suggested as a therapy for DME which is ineffective with laser treatment, particularly when macula traction does exist (such as vitreous macular traction, detachment between the preretinal membrane and traction retinal close to the macula) (263, 265).

As a pathogenic mediator of the abnormal growth and leakage of retinal blood vessels, VEGF has always been a treatment target for DR. The concentration of VEGF in the eye is closely related to hypoxia and active neovascularization. After laser photocoagulation, its concentration decreases, and VEGF inhibitor active drugs can improve ischemia-induced animal retinal neovascularization and prevent or even realize the reversal of PDR and DME (268, 269). A random trial contrasted laser treatment + intravitreous ranibizumab injection with laser treatment + placebo injection in DME sufferers (268). In the first year, the ranibizumab group gained approximately single-line extra vision compared to laser treatment. The frequency of vision improvement in the ranibizumab group was twice that of the laser group. Notably, in contrast to the eyes mainly exposed to laser treatment alone, the eyes exposed to laser and ranibizumab treatment are less probably to show significant vision loss (268). Diabetic individuals suffer more risk of cataracts that require surgical treatment. Nevertheless, in certain sufferers, cataract surgery could aggravate macula edema and retinopathy. As a result, sometimes, it may be necessary to perform laser therapy on DR sufferers before cataract surgery or immediately posterior to cataract surgery (270). Despite the fact that antiVEGF treatment has bright clinical use prospects in DR, its longterm security in diabetic sufferers hasn’t been determined. Local adverse incidents of intravitreous antiVEGF treatment involve cataract formation, amotio retinae, intravitreous hemorrhages, infections, and possible loss of neurally formed retina cells. Because of the relatively long halflife of certain drugs and the requirement for repeated administration, if a patient are exposed to anti-VEGF drugs for a long time, the risk of systemic vascular complications in him will increase, such as stroke and non-ocular (such as stomach and kidney) bleeding (109). In addition, most anti-VEGF drugs injected into the eyes may enter the systemic circulation and endanger key blood vessels, leading to ischemia. Other systemic adverse effects involve high blood pressure, albuminuria, and damaged wound repair (271).

Inflammatory events are significant for the etiopathogenesis of DR. Similar to antiVEGF drugs, intraocular corticosteroids are broadly utilized to heal DME (165, 260, 263, 272–274). Meta-analysis results have revealed that macular edema acting intravitreal triamcinolone acetonide (long-acting corticosteroid) can moderately improve vision (272, 273). A few clinical trials about longacting steroid implants (fluocinolone or dexamethasone) have also exhibited shortterm vision improvement (274).

## 7 Summary

**Figure 1:**
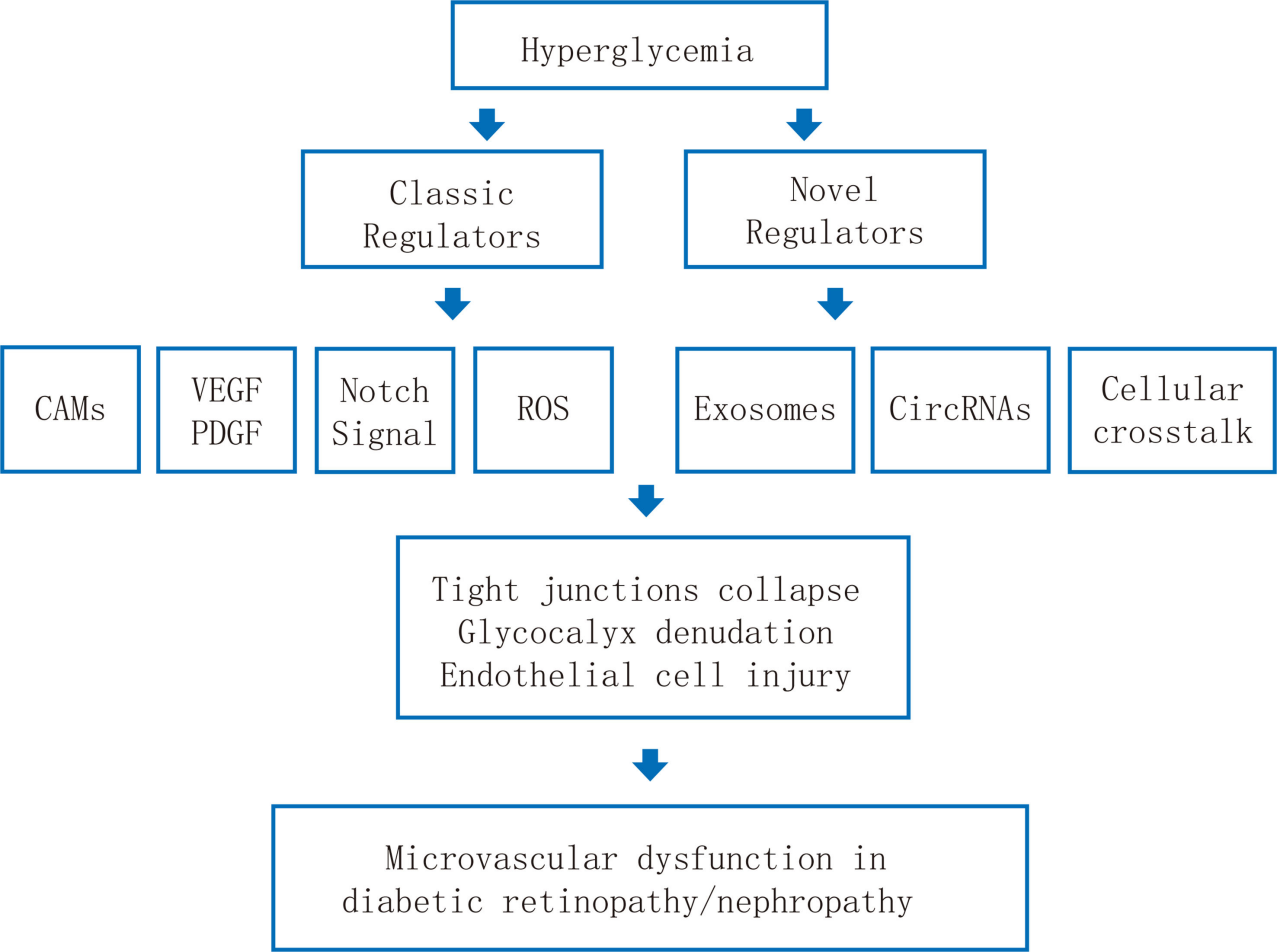
Schematic illustration of the pathological mechanisms of endothelial dysfunction in diabetic retinopathy and diabetic nephropathy.

The EC barrier is essential for maintaining the normal physiological activities of kidneys and retinas and excreting abnormal protein molecules and fluids. For diabetes, high glucose impairs both the retina and glomerular microvascular endothelium. This article elucidates the pathophysiology, some pathogenic sites and biochemical/cellular pathways of the retina and kidney microvascular endothelial injury in the diabetic microenvironment (**Figure 1**). The key molecular pathways causing DR and DN when the damage of retina and glomerulus microvascular endothelial barrier occurs mainly include (1): Hyperglycemia causes retinal ischemia and hypoxia, oxidative stress, inflammatory cell/factor accumulation, and activation of various metabolic pathways, leading to the destruction of the endothelial barrier structure and the increase of microvascular permeability, facilitating the incidence of DR and DN.

(2) The retina, kidney, and circulation are rich in exosomes. High glucose leads to changes in the number and content of exosomes, thereby causing the dysfunction of target endothelial cells in the retina and kidney.

(3) circRNA is abundant in the retina, kidney and circulation. It can act as a competitive endogenous RNA to sponge target miRNA, thereby affecting mRNA expression in the ECs of the retina and kidney and promoting the pathogenesis of DR and DN.

(4) Endothelial cells in the retina and kidney can interact with neighboring cells (such as renal tubular cells, pericytes) to generate diabetic endothelial dysfunction through intercellular communication and participate in the occurrence of DR and DN. Further researches on the retina and renal endothelial barrier functions would unravel DR and DN, hence giving birth to new biomarkers, therapeutic targets, or treatments eventually.

## Author Contributions

ZL conceptualized the ideas. JY performed the literature search, drafted the original manuscript, and drew the figures. All the authors approved the final version of the manuscript.

## Funding

This study is supported by grants from: 1. General Program of the National Natural Science Foundation of China General Project (No.81970633); 2. the Major public welfare special projects in Henan Province (No.201300310600); 3. The National Natural Science Young Scientists Foundation of China (No.81800648) 4. Excellent Young Scientists Fund Program of the Natural Science Foundation of Henan Province (No.202300410363). 5. Henan Province Medical Science and Technology Research Program Joint Construction Project (LHGJ20200337).

## Conflict of Interest

The authors declare that the research was conducted in the absence of any commercial or financial relationships that could be construed as a potential conflict of interest.

## Publisher’s Note

All claims expressed in this article are solely those of the authors and do not necessarily represent those of their affiliated organizations, or those of the publisher, the editors and the reviewers. Any product that may be evaluated in this article, or claim that may be made by its manufacturer, is not guaranteed or endorsed by the publisher.

## Glossary

**Table:**

AGEs	advanced glycation end products
GBM	glomerular basement membrane
ESL	endothelial surface layer
BRB	blood-retinal barrier
iBRB	inner BRB
AJs	adherence junctions
DN	diabetic nephropathy
GAG	glycosaminoglycans
T2D	type 2 diabetes
ESRD	end-stage renal disease
GFR	glomerular filtration rate
RPE	retinal pigment epithelium
NPDR	non-proliferative diabetic retinopathy
oBRB	outer BRB
RMECs	retinal microvascular endothelial cells
DME	Diabetic Macular Edema
VEGF	vascular endothelial growth factor
GJs	gap junctions
IL-6	interleukin 6
CAM	cell adhesion molecule
T1D	type 1 diabetes
PECAM-1	platelet/endothelial cell adhesion molecule-1
ROS	reactive oxygen species
LFA-1	Lymphocyte Function-Associated Antigen 1
PDR	proliferative diabetic retinopathy
eNOS	endothelial nitric oxide synthase
ELM	external limiting membrane
PDGF	platelet-derived growth factor
GEC	glomerular endothelial cell
MHC	major histocompatibility complex
Arg1	arginase 1
PCDR	Pre-clinical diabetic retinopathy
circRNA	circular RNA
HO-1	heme oxygenase-1
TLR4	toll-like receptor 4
HRMEC	human retinal microvascular EC
TECs	tubular epithelial cells
Ang-1	angiopoietin-1
Tie-2	tyrosine kinase 2
HIF-1&alpha;	hypoxia-inducible factor-1&alpha;
PC	pericytes
VEGFR-1	VEGF receptor-1
ECM	extracellular matrix
KDOQI	Kidney Disease Prognosis Quality Initiative
ICAM-1	intracellular adhesion molecule-1
JNC-8	Eighth Joint National Committee
ACE	angiotensin-converting enzyme
NP1	neuropilin-1
SGLT2	sodium-glucose cotransporter 2
BBB	blood-brain barrier
AA	amino acid
EC	endothelial cells
PRP	pan-retinal photocoagulation
PKC	protein kinase C
ALB	albumin
IV-C	collagen IV
BAB	blood-aqueous barrier
GF	growth factor
NP2	neuropilin-2
RBC	red blood cell
BG	blood glucose
ARB	angiotensin receptor blockers
SBP	Systolic Blood Pressure
DBP	diastolic blood pressure
JAK-1	Janus kinase-1
VCAM-1	vascular cell adhesion protein-1
KDIGO	Kidney Disease Prognosis Global Organization
MRA	mineralocorticoid receptor antagonist
TRD	traction retinal detachment
ETAR	Endothelin A Receptor
